# On Conduction in a Bacterial Sodium Channel

**DOI:** 10.1371/journal.pcbi.1002476

**Published:** 2012-04-05

**Authors:** Simone Furini, Carmen Domene

**Affiliations:** 1 Physical and Theoretical Chemistry Laboratory, Department of Chemistry, University of Oxford, Oxford, United Kingdom; 2 Department of Medical Surgery and Bioengineering, University of Siena, Siena, Italy; UNC Charlotte, United States of America

## Abstract

Voltage-gated Na^+^-channels are transmembrane proteins that are responsible for the fast depolarizing phase of the action potential in nerve and muscular cells. Selective permeability of Na^+^ over Ca^2+^ or K^+^ ions is essential for the biological function of Na^+^-channels. After the emergence of the first high-resolution structure of a Na^+^-channel, an anionic coordination site was proposed to confer Na^+^ selectivity through partial dehydration of Na^+^ via its direct interaction with conserved glutamate side chains. By combining molecular dynamics simulations and free-energy calculations, a low-energy permeation pathway for Na^+^ ion translocation through the selectivity filter of the recently determined crystal structure of a prokaryotic sodium channel from *Arcobacter butzleri* is characterised. The picture that emerges is that of a pore preferentially occupied by two ions, which can switch between different configurations by crossing low free-energy barriers. In contrast to K^+^-channels, the movements of the ions appear to be weakly coupled in Na^+^-channels. When the free-energy maps for Na^+^ and K^+^ ions are compared, a selective site is characterised in the narrowest region of the filter, where a hydrated Na^+^ ion, and not a hydrated K^+^ ion, is energetically stable.

## Author Summary

Ion channels are integral membrane proteins that control the passive diffusion of ions down their electrochemical gradient. According to the most permeating ion species, ion channels are classified in three categories: K^+^-channels, Na^+^-channels, and Ca^2+^-channels. The atomic structure of a K^+^-channel was the first to be solved experimentally more than 10 years ago. This structure inspired numerous computational studies, which revealed the mechanisms of conduction and selectivity in K^+^-channels. Recently, the first atomic structure of a Na^+^ selective channel has been solved. Here, molecular dynamics simulations and free-energy calculations are described and a possible mechanism for Na^+^ conduction is identified. In contrast to what it is observed in K^+^-channels, ion movements through Na^+^-channels appeared highly uncorrelated.

## Introduction

Sodium channels allow the passive diffusion of Na^+^ ions down their electrochemical gradient. They were first discovered, together with potassium selective channels, in nerve fibres where they mediate the fast depolarizing phase of action potentials [1]. Na^+^-channels are also involved in the initiation of action potentials in cardiac myocytes and in general, in the propagation of electrical impulses in cardiac, muscle and nerve cells. The cell membrane is exposed to a high-Na^+^/low-K^+^ concentration on the extracellular side, and to a low-Na^+^/high-K^+^ concentration on the intracellular side. It is the selectivity of Na^+^ and K^+^ channels, in the presence of these concentration gradients what makes possible the control of the membrane potential. Several atomic structures of K^+^-selective channels have been solved since 1998 [2], [3], [4], [5], [6]. Recently, the atomic structure of a Na^+^-selective channel, NavAb from the bacterium *Arcobacter butzleri*, has also been determined [7], and provides the opportunity to investigate how selectivity and conduction are realized at the atomic level in Na^+^-channels.

**Figure 1:**
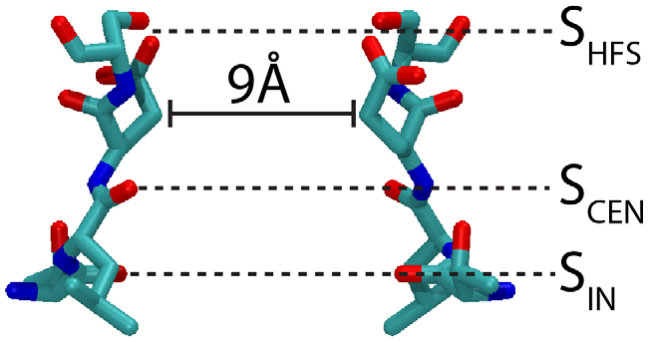
Structure of the selectivity filter of NavAb. Residues 175 to 178 (TLES) of two opposite subunits are shown in licorice representation. S_IN_, S_CEN_ and S_HFS_ indicate possible ion binding sites. The distance between the C_β_ atoms of two Glu177 residues is indicated.

Na^+^ and K^+^-channels share the same general architecture, with four transmembrane domains contributing to a central pore. This central pore, where ion permeation occurs, is delimited by two transmembrane helices, S5 and S6, linked by an intervening loop. The Na^+^-channel NavAb was crystallized in the closed state, where the four S6 helices are arranged in a conical shape, defining a bundle-crossing at the intracellular side and a water-filled cavity above it. In the open state, the S6 helices are thought to bend, and the water-filled cavity becomes a continuous with the intracellular solution, as observed in K^+^-channel [8], [9]. The region responsible for selective permeation is located in the loop between S5 and S6. In K^+^-channels, each chain contributes to the selectivity filter with the signature sequence T/S-x-G-Y/F-G. The oxygen atoms of the residues from this signature sequence define a series of four binding sites where dehydrated K^+^ ions bind [2]. The selectivity filter of the bacterial Na^+^-channel NavAb is much wider than that of known K^+^-channels (Figure 1). Although Na^+^ ions were not observed in the crystallographic structure, the presence of three Na^+^ binding sites was hypothesized [7]. Residues Thr175 and Leu176 define two rings of carbonyl oxygen atoms at the intracellular entrance of the filter. Water molecules from the hydration shell of a Na^+^ ion may interact through hydrogen bonds with these two layers of carbonyl oxygen atoms, thus defining two binding sites for hydrated Na^+^ ions. Following the convention introduced by Payandeh et al [7], these binding sites will be referred as S_IN_ and S_CEN_. A third Na^+^ binding site, S_HFS_, composed by side chains of four glutamate residues, Glu177, from each of the four protein chains was also proposed [7]. The side chain of Glu177 points to the lumen of the filter, and they could contribute to the hydration shell of cations.

Molecular Dynamics (MD) simulations were crucial for understanding the mechanisms of conduction and selectivity in K^+^-channels. The knock-on mechanism, first hypothesized in the fifties by Hodgkin and Keynes [10], was described at atomic detail by computational studies [11], [12], and free-energy calculations revealed energy barriers of the order of 2–4 kcal/mol for the concerted motion of three K^+^ ions through the filter of K^+^-channels [13], [14], [15]. These free-energy barriers along a multi-ion permeation pathway were shown to increase if one of the permeating ions was Na^+^ instead of K^+^, offering some insight about selectivity in K^+^-channels [16], [17], [18]. The recent crystal structure of a prokaryotic voltage gated sodium channel NavAb from *Arcobacter butzleri* in combination with all-atom MD simulations can also provide important information concerning conduction and selectivity in Na^+^-channels. Here, simulation strategies similar to the ones used for K^+^-channels were adopted to analyze conduction and selectivity in the NavAb Na^+^-channel, with the purpose to get insights into: (i) the number of Na^+^ binding sites, (ii) the nature of the conduction mechanism, and (iii) selectivity.

## Results

Root mean square deviation of the protein backbone atoms below 1.4 Å, and 1.0 Å in the case of the backbone atoms of the filter, residue Thr175 to Ser178, were observed in a 40-ns MD trajectory, which suggests that the system is stable. The selectivity filter was initially occupied by a single Na^+^ ion in S_CEN_, which remained at this position for the entire trajectory. A second Na^+^ ion approached the filter from the extracellular side during the course of the simulation, and it stayed close to S_HFS_ for more than 10 ns. The intracellular cavity below the selectivity filter was initially occupied by 15 water molecules. The number of water molecules inside the cavity reached equilibrium value of ∼45 in the course of the simulation, due to the incorporation of water molecules from the extracellular solution across the selectivity filter. This contrasts with the situation in K^+^-channels, where the movement of water molecules through the selectivity filter was highly unlikely [19]. Similar observations where described by Klein et al [20].

**Figure 2:**
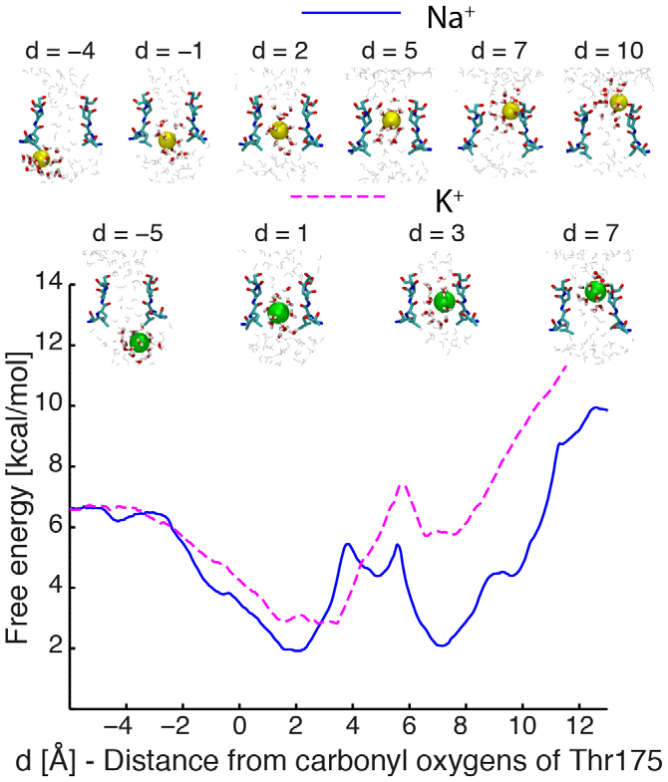
Potential of Mean force for conduction events with one Na^+^ ion and one K^+^ ion. Values on the x-axis correspond to the distance between the permeating ion and the centre of the carbonyl oxygen atoms of residues Thr175 along the pore axis. The free-energy for a permeating Na^+^ ion is shown in a blue continuous line and K^+^ in a pink dashed line. Snapshots from the umbrella sampling trajectories are shown for some significant configurations. Residues 175 to 178 of two opposite subunits are depicted in licorice representation, together with the permeating ion in VDW spheres, yellow and green for Na^+^ and K^+^. Water molecules closer than 5 Å (licorice representation) and 15 Å (grey lines) from the ion are also shown.

MD simulations in the nanosecond time scale cannot reveal the mechanisms of conduction and selectivity in Na^+^-channels alone. In order to get further insights into the permeation process, the permeation free-energy profiles for Na^+^ and K^+^ ions in the Na^+^-channel were calculated by the umbrella sampling technique (see the Methods section for the computational details). The free-energy profile of a single Na^+^ ion moving from the intracellular cavity to the extracellular solution displays two minima with similar energies (Figure 2). In the innermost minimum, the ion lies in the plane defined by the carbonyl oxygen atoms of residues Leu176, binding site S_CEN_. Figure S1 shows the positions of the permeating ion in a plane perpendicular to the permeation axis. At S_CEN_, a Na^+^ ion preserves its entire hydration shell, i.e. it is coordinated only by oxygen atoms from the surrounding water molecules. The nature of the atoms of the coordination shell of a Na^+^ ion at different positions along the permeation pore is shown in Figure S2. In the region above S_CEN_, the permeating ion may move ∼2 Å away from the pore axis, where it interacts with a carbonyl oxygen atom of Leu176 from one of the chains. This position does not correspond to a minimum in the free-energy profile. Proceeding toward the extracellular side, a local free-energy minimum is encountered with the ion close to the pore axis, in a region 1–2 Å below the oxygen atoms of the side chain of Glu177. At this position, the Na^+^ ion is fully solvated by water molecules. This local minimum is followed by the second global free-energy minimum, where the ion occupies S_HFS_. The ion moves away from the centre of the pore axis (Figure S1) and two of the water molecules from the hydration shell are lost, and substituted by oxygen atoms from the side chain of Glu177 and Ser178 (Figure S2).

**Figure 3:**
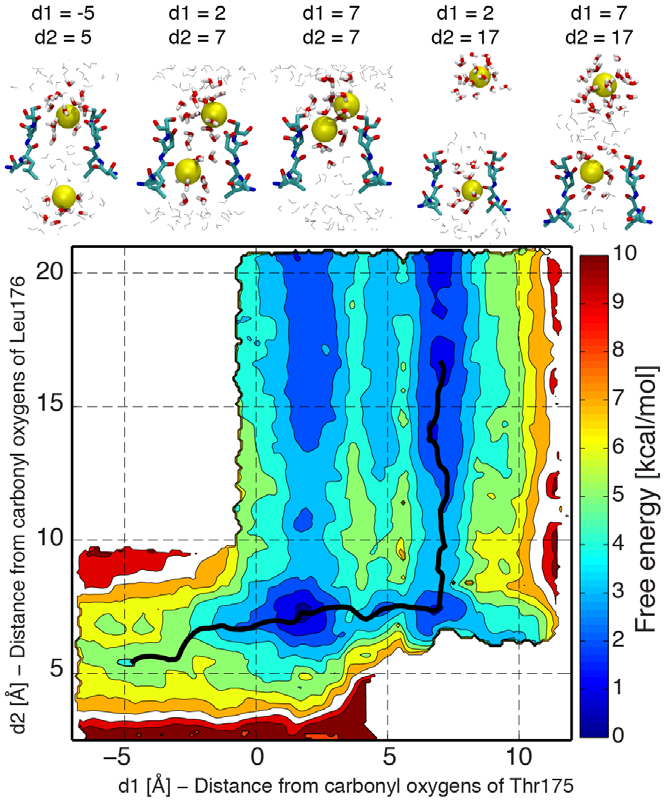
Potential of Mean force for conduction events with two Na^+^ ions. Values on the x/y-axis correspond to distances along the pore axis between the permeating ions and the centre of the carbonyl oxygen atoms of residues Thr175 (d1) and Leu176 (d2) respectively. Counter lines are drawn every 1 kcal/mol. Snapshots from the umbrella sampling trajectories are shown for some significant configurations. Residues 175 to 178 of two opposite subunits are depicted in licorice representation, together with the permeating Na^+^ ion (yellow). Water molecules closer than 5 Å (licorice representation) and 15 Å (grey lines) to the ion are shown. The minimum energy path between a minimum with one ion in the cavity and a minimum with one ion in extracellular solution is shown as a black line. The free energy along the minimum energy path is shown in Figure S3.

The free-energy profile of a single K^+^ ion permeating the selectivity filter of the Na^+^-channel is markedly different from the free-energy profile of single Na^+^ ion (Figure 2). In the most stable minimum, a K^+^ ion occupies a position similar to the one occupied by Na^+^ in its innermost minimum. As observed with Na^+^, a K^+^ ion at this position preserves intact its hydration shell. The second minimum is analogous to the outermost minimum encountered by Na^+^, with oxygen atoms from the side chains of Glu177 and Ser178 contributing to the coordination shell of the ion. In contrast to Na^+^, no local minimum is present in the region between the carbonyl oxygen atoms of Leu176 and the side chain oxygen atoms of Glu177 in the permeation pathway of K^+^. Overall, the composition of the coordination shell and the position of the ion in a plane perpendicular to the pore axis are analogous for K^+^ and Na^+^ ions (Figure S1 and S2). Likewise, a free-energy barrier higher than 8 kcal/mol hampers the movement of both Na^+^ and K^+^ ions from S_HFS_ to the extracellular solution. These high free-energy barriers associated with the exit of Na^+^ and K^+^ from the selectivity filter may originate from the binding affinity for cations of this region of the pore, where the carboxyl groups of the side chain of Glu177 line the pore on the extracellular side. Therefore, under these circumstances, to enhance conduction, it seems possible that the selectivity filter is occupied on average by one or more positive ions, and that conduction occurs when an incoming ion displaces the ion(s) already inside the pore, like described in K^+^-channels [14]. To test this hypothesis, free-energy maps for conduction events with two Na^+^ ions were calculated (Figure 3). It was found that in the most stable configuration, both S_CEN_ and S_HFS_ are occupied by Na^+^ ions. Starting from this position and moving the innermost ion toward the intracellular side, a local minimum is observed with the bottom ion inside the cavity and the top ion in S_HFS_. The free energy of this local minimum is 4.3±0.4 kcal/mol higher than the free-energy of the minimum with ions in S_CEN_ and S_HFS_. The differences in free energy and the energy barriers were evaluated along the minimum energy path that connects the local energy minima, and the errors were estimated dividing the trajectories into three data sets (see the Methods section for further details). A second free-energy minimum is observed with both ions close to Glu177 and Ser178. The free energy of this configuration is 1.2±0.6 kcal/mol higher than the free energy of the minimum with ions in S_CEN_ and S_HFS_. The free-energy barriers between the two basins are 3.5±0.5 kcal/mol and 2.4±0.3 kcal/mol respectively in the outward and inward directions. Regardless of the position of the bottom ion (S_CEN_ or S_HFS_), the movement between the extracellular solution and the pore of the outermost ion does not encounter any free-energy barriers higher than 3 kcal/mol.

**Figure 4:**
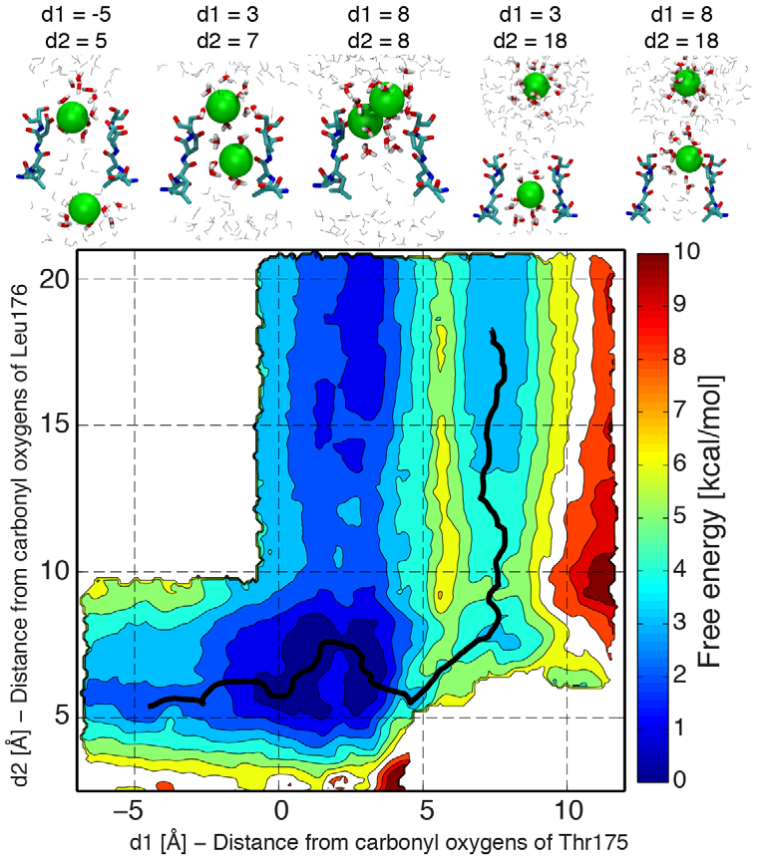
Potential of Mean force for conduction events with two K^+^ ions. K^+^ ions are shown in green. Legend as in Figure 3.

In order to understand if the low free-energy barriers for ion translocation across the pore were exclusive of Na^+^ ions, free-energy maps for conduction events with two K^+^ ions were also calculated (Figure 4). Like in the case of Na^+^ ions, the lowest free-energy configuration has two ions occupying binding sites S_CEN_ and S_HFS_, but in contrast to a pore occupied by Na^+^ ions, this free-energy minimum is much broader. A local minimum with both ions close to Glu117 and Ser178 can be also described. The free energy of this configuration is 2.7±0.5 kcal/mol higher than the free energy of the lowest minimum, and the barriers between the two basins are 5.0±0.4 kcal/mol and 2.3±0.5 kcal/mol respectively in the outward and inward directions. The inward movement of the ion at S_CEN_ toward the intracellular cavity while the second ion is at S_HFS_ causes an energy increase of 1.8±0.2 kcal/mol. The change in free energy is lower than 1 kcal/mol if the ion in S_HFS_ leaves the filter toward the extracellular solution and the barriers for the entry/exit of K^+^ in S_IN_ and S_HFS_ are below 2 kcal/mol.

**Figure 5:**
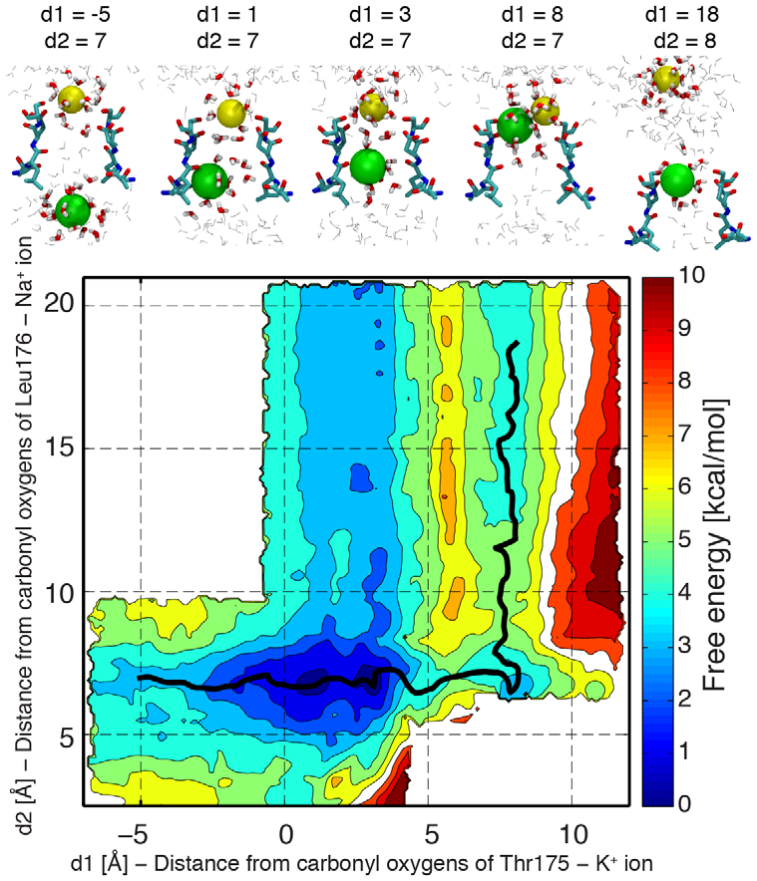
Potential of Mean force for conduction events of a mixture of one K^+^ and one Na^+^ ion. K^+^ and Na^+^ ions are shown in green and yellow respectivelly. Legend as in Figure 3.

Under physiological conditions, the cellular membrane is exposed to high-Na^+^/low-K^+^ concentration on the extracellular side, and to a low-Na^+^/high-K^+^ concentration on the intracellular side. As a consequence, the selectivity filter of Na^+^-channels is more likely to be approached by Na^+^ ions on the extracellular side and by K^+^ ions on the intracellular side. To characterize this situation, the free-energy map for a mixture of K^+^ and Na^+^ ions in the selectivity filter was calculated, with the K^+^ ion in the innermost position and the Na^+^ ion in the outermost position (Figure 5). The free-energy profile characterising the outward movement of K^+^ through the selectivity filter is similar to that when both ions are K^+^. In the lowest free-energy configuration, the Na^+^ ion is in site S_HFS_, while the K^+^ ion can move in a wide region around S_CEN_, experiencing low-energy barriers (<2 kcal/mol). A second local minimum exists with both ions close to Glu177 and Ser178 (2.6±0.8 kcal/mol higher in energy). The barriers between the two minima are similar to those observed in the case of two K^+^ ions (4.6±1.1 in the outward direction, 2.0±0.5 in the inward direction).

## Discussion

Since the number and position of Na^+^ binding sites in the NavAb channel were only speculated using the crystallographic structure, insight from atomistic simulations is essential. Two Na^+^ binding sites have been described in the selectivity filter in simulations with either one or two permeating ions. These binding positions correspond to sites S_CEN_ and S_HFS_, which were hypothesized experimentally. Computations confirm that a Na^+^ ion remains close to the centre of the pore axis and it is fully hydrated in the innermost binding site. In contrast, it occupies a position slightly off the centre of the pore axis, while it interacts directly with the side chain of Glu177 in the outermost site. The side chain oxygen atom of Ser178 also contributes to the coordination shell of a Na^+^ ion in S_HFS_, which was not an obvious experimental observation. The experimentally speculated binding site S_IN_ does not appear as a well defined minimum in the free-energy maps from atomistic simulations. A configuration with two ions in S_CEN_ and S_HFS_ is found to be the most stable during conduction events with two permeating ions. Surprisingly, the energetic cost of having two Na^+^ ions in close proximity of binding site S_HFS_ is extremely low (∼1 kcal/mol). Therefore, the picture that emerges from the free-energy profiles is that of a pore preferentially occupied by two ions, which can switch between different configurations by crossing low energy-barriers. In contrast to K^+^-channels, the movements of the ions appear to be weakly coupled.

The nature of the conduction mechanism for K^+^ ions in this Na^+^-channel was also tested, in order to understand selectivity. In the case of Na^+^, the strongest impediment to conduction is the raise in free energy associated with the inward movement of an ion from S_CEN_ to the intracellular cavity. Similarly, the free energy also increases when a K^+^ ion moves from S_CEN_ to the intracellular cavity, but to a lower extent (∼2 kcal/mol for K^+^; ∼4 kcal/mol for Na^+^). The free energy of an ion in the cavity is likely to be affected by the conformation of the intracellular gate in NavAb, which is closed. And this effect may be different for K^+^ and Na^+^, as already observed in K^+^-channels [21].

The biological function of Na^+^-channels is to allow Na^+^ ions inside the cell at nearly the rate of free diffusion, while at the same time blocking the flux of K^+^ ions in the outward direction. During permeation in the selectivity filter, the highest free-energy barrier that a Na^+^ ion experiences in the inward direction is ∼2 kcal/mol (independently of the nature of the innermost ion, Na^+^ or K^+^), while the highest energy barrier for the outward movement of K^+^ ions is ∼5 kcal/mol (independently of the nature of the outermost ion, Na^+^ or K^+^). Considering that discrimination of ions is performed with an efficiency of one K^+^ ion every 10–100 Na^+^ ions [7], [22], the differences in free-energy barriers of 1–2 kcal/mol described before are perfectly in line with the experimental observations. The highest barrier for the passage of ions through the filter is localized between S_CEN_ and S_HFS_, both for K^+^ and Na^+^. In the case of Na^+^, a local free-energy minimum exists in this region, with the ion fully hydrated and aligned at the centre of the channel axis. In contrast, the same configuration is not a free-energy minimum for K^+^. This difference is already evident in the free-energy profiles with a single permeating ion. The instability of a hydrated K^+^ ion in the middle of the selectivity filter is a possible cause of the preference of the channel for Na^+^ permeation. The shortcomings of the adopted computational methods should be clearly kept in mind when interpreting the present results. The convergence of the PMF calculations in complex molecular systems is already a well reported issue [23], [24]. Here, the magnitude of the errors of the PMF was calculated by comparing different parts of the simulated trajectories. This approach is reliable if the simulated trajectories are extensive enough to sample the relevant ion configurations in the selectivity filter. However, the possibility that structural changes of the protein on a longer time scale may affect the calculated energy profiles cannot be ruled out. In contrast to K^+^-channels, the movements of the ions appear to be weakly coupled in Na^+^-channels, and the ions maintain their hydration shell. Therefore, the issue of the absolute binding energy values of the ions [11], that is, the energy of the ions in the protein relative to their energy in water should be less crucial than in the case of K^+^-channels. In the later, the water molecules of the ion's hydration shell are replaced with the carbonyl oxygen atoms of the protein. A particular critical point is the presence of ionisable residues in the close proximity of the permeation pathway (Glu177). The ionization state of these residues may have an important effect on the permeation properties, and need to be closely analyzed. In this respect, it is also important to remember that a non-polarisable force field has been used and polarization effects may be important in the present context [25], [26], [27]. Such calculations will be reported in the future.

Another crucial point in this type of studies that was clearly pointed out by Warshel et al. [28] is that ‘the issue of ion selectivity cannot be resolved quantitatively without calculating the corresponding ion current, which is the actual direct observable of the system’. As they explained in their paper ‘such a calculation should be able to convert the free energy profile of the system to corresponding time dependence of the ion permeation process’. In order to do this the present study should be extended, and the free energy obtained here should be coupled to Brownian dynamics simulations as those reported by Warshel et al. [28]. In such a way, under certain conditions that allow replicating experimental data and using the calculated free energy profiles as starting point, a detailed understanding of ion selectivity may then be possible.

## Methods

### Model Definition

Atomic coordinates of the NavAb channel were taken from the Protein Data Bank entry 3RVY [7]. Only residues 116 to 221 that correspond to the pore region of the channel were included in the model. Default protonation states were used for all the ionisable residues. N- and C-terminals were amidated and acetylated respectively. The channel was centred in the x-y plane and the permeation pathway was aligned with the z-axis. The aromatic belt defined by the amphipathic residues Trp195 was aligned with the upper layer of a pre-equilibrated bilayer of DOPC molecules. All lipid molecules closer than 2.0 Å to the protein atoms were removed. The system was solvated by ∼20.000 water molecules, and 32 Na^+^ ions and 24 Cl^−^ ions were added. A Na^+^ ion was positioned in site S_CEN_. Harmonic restraints were initially applied to the backbone atoms and to the Na^+^ ion in the selectivity filter, and gradually removed during a one nanosecond period. The total production run was 40 ns.

MD trajectories were simulated with the version 2.7 of NAMD [29], using the CHARMM27 force field with CMAP corrections [30], and the TIP3P model for water molecules [31]. Standard parameters for K^+^ and Na^+^ in CHARMM27 force field were adopted. Simulations were performed in the NpT ensamble. Pressure was kept at 1atm by the Nose-Hoover Langevin piston method [32], [33], with a damping time constant of 100 ps and a period of 200 ps. Temperature was kept at 300 K by coupling to a Langevin thermostat, with a damping coefficient of 5 ps^−1^ [33]. Electrostatic interactions were treated by the Particle Mesh Ewald algorithm, with grid spacing below 1 Å [34]. Van der Waals interactions were truncated at 12 Å, and smoothed at 10 Å. Hydrogen atoms were restrained by the SETTLE algorithm [35], which allowed a 2 fs time-step.

### Free-Energy Calculation

Free-energy profiles for one and two ion conduction events were calculated by umbrella sampling [36]. The reaction coordinate for one ion conduction events was the distance along the z-axis between the permeating ion and the centre of the carbonyl oxygen atoms of Thr175. The same reaction coordinate was used for the bottom ion in the analyses of conduction events considering two ions. The reaction coordinate for the upper ion was the distance along the z-axis between the ion and the centre of the carbonyl oxygen atoms of Leu176. Harmonic potentials (force constant 10 kcal*mol^−1^*Å^−2^) were applied to the reaction coordinates. Each umbrella sampling simulation consisted of 0.5 ns, and the first 50 ps were considered as equilibration period and discarded. 20 and 270 umbrella sampling simulations were performed respectively for conduction events considering one or two ions, moving the centres of the harmonic potentials in 1.0 Å steps. The starting configurations for the umbrella sampling simulations were defined using the final snapshot of the normal MD trajectory. The water oxygen atoms or the Na^+^ ions closer to the centres of the reaction coordinates were selected. Then, the positions of the ions subjected to the harmonic potentials were switched with the positions of these molecules/ions closer to the centre of the harmonic potentials. The ions restrained in the umbrella sampling simulations were the ion already inside the selectivity filter in the normal MD simulation, plus an ion randomly taken from the extracellular solution in the case of two-dimensional umbrella sampling simulations. For simulations with K^+^ ions, all the Na^+^ ions were alchemically transformed to K^+^ ions in the first snapshot, while for the calculation of the energy map for Na^+^/K^+^ mixtures, only one ion (the innermost of the two restrained ions) was transformed to K^+^. Free-energy profiles were calculated by the Weighted Histogram Analysis Method [37]. The string method [38] was used to calculate the minimum energy path in free-energy profiles of conduction events with two ions (Figure S3). The initial guess for the minimum energy path was the segment that connects the free-energy minimum with the innermost ion in the cavity, and the free-energy minimum with the outermost ion in the extracellular solution (200 equally spaced points were used for the discretization). The path evolved in the direction opposite to the free-energy gradient until the root mean square distance between two successive iterations was below 10^−3^ Å. The relative energies of the local minima and the energy barriers between different free-energy basins provided in the text are evaluated along this minimum energy path. In order to estimate the errors affecting the free-energy values, the umbrella sampling trajectories were divided into 3 separate sets of 150 ps each. Free-energy profiles and minimum energy paths were computed separately for each data set. The values reported in the text are the average and the standard deviation among these 3 data sets. The free-energy profiles and the minimum energy paths shown in Figures 2, 3, 4, 5 and S3 were calculated using the whole umbrella sampling trajectories after the equilibration period.

Note Added In Proof. While this paper was in revision similar results were reported by B. Corry and M. Thomas in J. Am. Chem. Soc., 2012, 134 (3), pp 1840–1846.

## Supporting Information

Figure S1 **Na^+^/K^+^ displacement in the x-y plane.** The displacement of Na^+^/K^+^ ions with respect to the channel axis is shown for an ion in different positions along the pore axis: (A) from the carbonyl oxygen atoms of Thr175 to those of Leu176, (B) from the carbonyl oxygen atoms of Leu176 to 3 Å above them, (C) from 3 Å above the carbonyl oxygen atoms of Leu176 to 1 Å below the side chain oxygen atoms of Glu177, and (D) from 1 Å below to 1 Å above the side chain oxygen atoms of Glu177. All the umbrella sampling trajectories from the simulations with two ions were considered for the analysis, taking snapshots every 20 ps after the equilibration period.
(TIF)

Figure S2 **Coordination number of Na^+^ and K^+^ ions.** Oxygen atoms were considered part of the coordination shell of the ion if closer than 2.8 Å from a Na^+^ ion or 3.2 Å from a K^+^ ion. All the umbrella-sampling trajectories from the simulations with two ions were used for this analysis, taking snapshots every 20 ps after the equilibration period. The black line shows the total number of coordinating oxygen atoms, which is the sum of the oxygen atoms coming from: water molecules (blue line), protein backbone (pink line), Glu177 side chain (red line), and Ser178 side chain (green line). The average positions along the filter axis of the carbonyl oxygen atoms of Thr175, Leu176, and Ser178 are shown along the x-axis.
(TIF)

Figure S3 **Free energy along the minimum energy path for Na^+^ ions (A), K^+^ ions (B), and K^+^/Na^+^ mixture with K^+^ in the innermost position (C).** The minimum energy path was calculated between a local free-energy minimum with the bottom ion in the intracellular cavity and the top ion inside the selectivity filter, and a local free-energy minimum with the bottom ion inside the selectivity filter and the top ion in the extracellular solution. The path was discretized using 200 equally spaced points, and the minimum energy path was calculated using the string method. The values of the reaction coordinates, d1 and d2, along the minimum energy path are shown along the bottom and top x-axis for a subset of the local free-energy minima. d1/d2 are defined as the distance along the pore axis between the permeating ion in the inward/outward position and the centre of the carbonyl oxygen atoms of residues Thr175/Leu176.
(TIF)

## References

[1] Hodgkin AL, Huxley AF (1952). Currents carried by sodium and potassium ions through the membrane of the giant axon of Loligo.. J Physiol.

[2] Doyle DA, Cabral JM, Pfuetzner RA, Kuo A, Gulbis JM (1998). The structure of the potassium channel: molecular basis of K^+^ conduction and selectivity.. Science.

[3] Jiang Y, Lee A, Chen J, Cadene M, Chait BT (2002). Crystal structure and mechanism of a calcium-gated potassium channel.. Nature.

[4] Jiang Y, Lee A, Chen J, Ruta V, Cadene M (2003). X-ray structure of a voltage-dependent K^+^ channel.. Nature.

[5] Kuo A, Gulbis JM, Antcliff JF, Rahman T, Lowe ED (2003). Crystal structure of the potassium channel KirBac1.1 in the closed state.. Science.

[6] Long SB, Campbell EB, Mackinnon R (2005). Crystal structure of a mammalian voltage-dependent Shaker family K^+^ channel.. Science.

[7] Payandeh J, Scheuer T, Zheng N, Catterall WA (2011). The crystal structure of a voltage-gated sodium channel.. Nature.

[8] Cuello LG, Romero JG, Cortes DM, Perozo E (1998). pH-Dependent gating in the *Streptomyces lividans* K^+^ channel.. Biochem.

[9] Jiang YX, Lee A, Chen JY, Cadene M, Chait BT (2002). The open pore conformation of potassium channels.. Nature.

[10] Hodgkin AL, Keynes RD (1955). The potassium permeability of a giant nerve fibre.. J Physiol.

[11] Burykin A, Schutz CN, Villa J, Warshel A (2002). Simulations of ion current in realistic models of ion channels: The KcsA potassium channel.. Proteins.

[12] Jensen MO, Borhani DW, Lindorff-Larsen K, Maragakis P, Jogini V (2010). Principles of conduction and hydrophobic gating in K^+^ channels.. Proc Natl Acad Sci U S A.

[13] Aqvist J, Luzhkov V (2000). Ion permeation mechanism of the potassium channel.. Nature.

[14] Berneche S, Roux B (2001). Energetics of ion conduction through the K^+^ channel.. Nature.

[15] Furini S, Domene C (2009). Atypical mechanism of conduction in potassium channels.. Proc Natl Acad Sci U S A.

[16] Thompson AN, Kim I, Panosian TD, Iverson TM, Allen TW (2009). Mechanism of potassium-channel selectivity revealed by Na^+^ and Li^+^ binding sites within the KcsA pore.. Nat Struct Mol Biol.

[17] Egwolf B, Roux B (2010). Ion selectivity of the KcsA channel: a perspective from multi-ion free energy landscapes.. J Mol Biol.

[18] Furini S, Domene C (2011). Selectivity and permeation of alkali metal ions in K+-channels.. J Mol Biol.

[19] Furini S, Beckstein O, Domene C (2009). Permeation of water through the KcsA K^+^ channel.. Proteins.

[20] Carnevale V, Treptow W, Klein ML (2011). Sodium ion binding sites and hydration in the lumen of a bacterial ion channel from molecular dynamics simulations.. J Phys Chem Lett.

[21] Zhou Y, MacKinnon R (2004). Ion binding affinity in the cavity of the KcsA potassium channel.. Biochemistry.

[22] Favre I, Moczydlowski E, Schild L (1996). On the structural basis for ionic selectivity among Na^+^, K^+^, and Ca^2+^ in the voltage-gated sodium channel.. Biophys J.

[23] Kato M, Warshel A (2005). Through the channel and around the channel: Validating and comparing microscopic approaches for the evaluation of free energy profiles for ion penetration through ion channels.. J Phys Chem B.

[24] Pohorille A, Jarzynski C, Chipot C (2010). Good practices in free-energy calculations.. J Phys Chem B.

[25] Illingworth CJR, Furini S, Domene C (2010). Computational studies on polarization effects and selectivity in K^+^ channels.. J Chem Theory Comp.

[26] Warshel A, Kato M, Pisliakov AV (2007). Polarizable force fields: History, test cases, and prospects.. J Chem Theory Comp.

[27] Illingworth CJ, Domene C (2009). Many-body effects and simulations of potassium channels.. Proc Roy Soc A.

[28] Burykin A, Kato M, Warshel A (2003). Exploring the origin of the ion selectivity of the KcsA potassium channel.. Proteins.

[29] Phillips JC, Braun R, Wang W, Gumbart J, Tajkhorshid E (2005). Scalable molecular dynamics with NAMD.. J Comp Chem.

[30] MacKerell AD, Bashford D, Bellott, Dunbrack RL, Evanseck JD (1998). All-atom empirical potential for molecular modeling and dynamics studies of proteins.. J Phys Chem B.

[31] Jorgensen WL, Chandrasekhar J, Madura JD, Impey RW, Klein ML (1983). Comparison of simple potential functions for simulating liquid water.. J Chem Phys.

[32] Martyna GJ, Tobias DJ, Klein ML (1994). Constant pressure molecular dynamcis algorithms.. J Chem Phys.

[33] Feller SE, Zhang YH, Pastor RW, Brooks BR (1995). Constant-pressure molecular dynamics simulation- the langevin piston method.. J Chem Phys.

[34] Essmann U, Perera L, Berkowitz ML, Darden T, Lee H (1995). A smooth particle mesh Ewald method.. J Chem Phys.

[35] Miyamoto S, Kollman PA (1992). Settle - An analytical version of the Shake and Rattle algorithm for wigid water molecules.. J Comp Chem.

[36] Torrie GM, Valleau JP (1977). Nonphysical sampling distributions in Monte-Carlo free energy distributions: umbrella sampling.. J Comp Phys.

[37] Kumar S, Bouzida D, Swendsen RH, Kollman PA, Rosenberg JM (1992). The weighted histogram analysis method for free-energy calculations on biomolecules .1. The method.. J Comp Chem.

[38] E WN, Ren WQ, Vanden-Eijnden E (2007). Simplified and improved string method for computing the minimum energy paths in barrier-crossing events.. J Chem Phys.

