# Supplementary figures and images for: On Conduction in a Bacterial Sodium Channel
Source: PLoS Comput Biol. 2012 Apr 5;8(4):e1002476. doi: 10.1371/journal.pcbi.1002476 (PMC3320569; doi:10.1371/journal.pcbi.1002476)

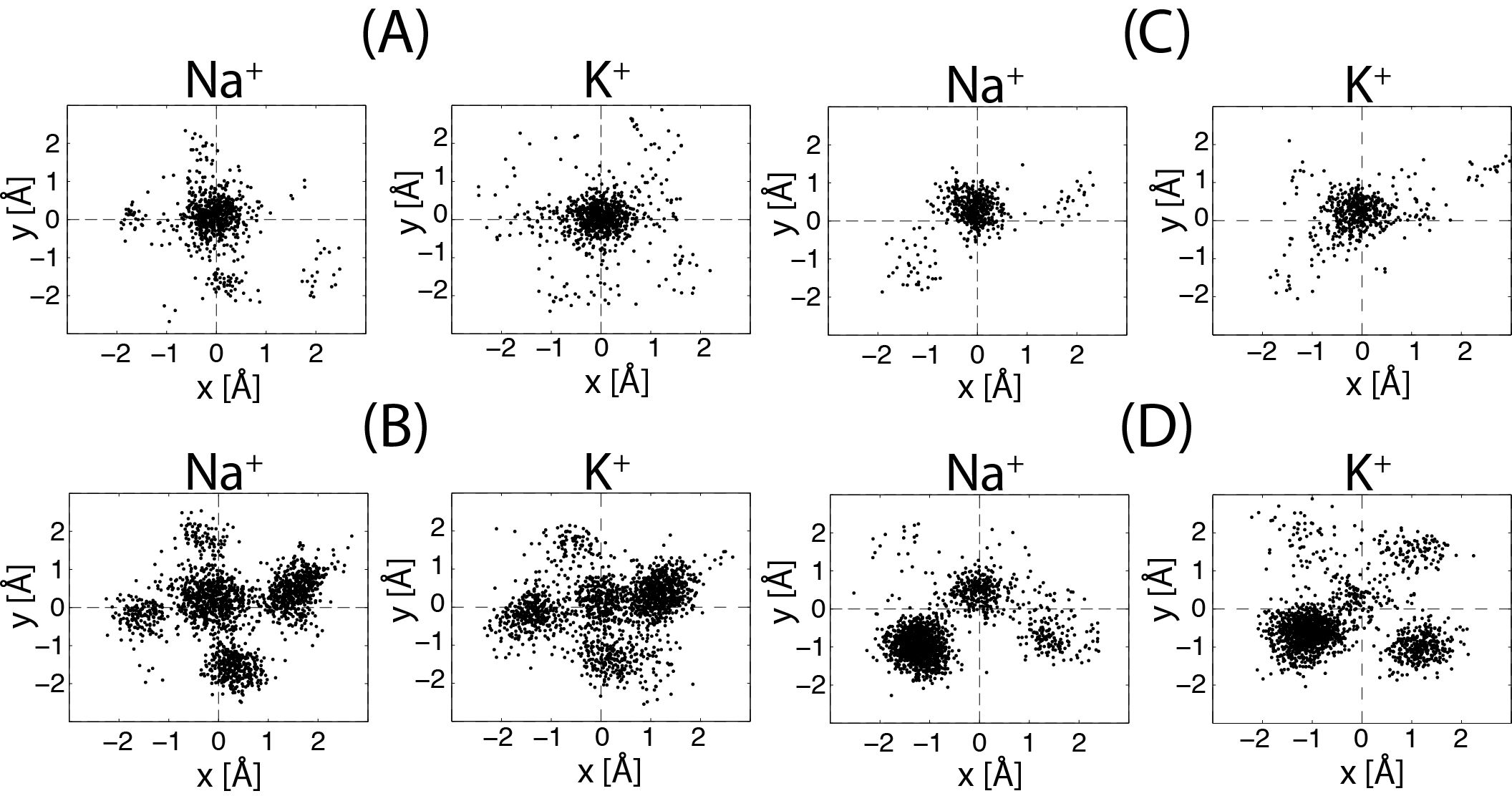

Supplement: Figure S1 — Na+/K+ displacement in the x-y plane. The displacement of Na+/K+ ions with respect to the channel axis is shown for an ion in different positions along the pore axis: (A) from the carbonyl oxygen atoms of Thr175 to those of Leu176, (B) from the carbonyl oxygen atoms of Leu176 to 3 Å above them, (C) from 3 Å above the carbonyl oxygen atoms of Leu176 to 1 Å below the side chain oxygen atoms of Glu177, and (D) from 1 Å below to 1 Å above the side chain oxygen atoms of Glu177. All the umbrella sampling trajectories from the simulations with two ions were considered for the analysis, taking snapshots every 20 ps after the equilibration period. (TIF) [file pcbi.1002476.s001.tif]

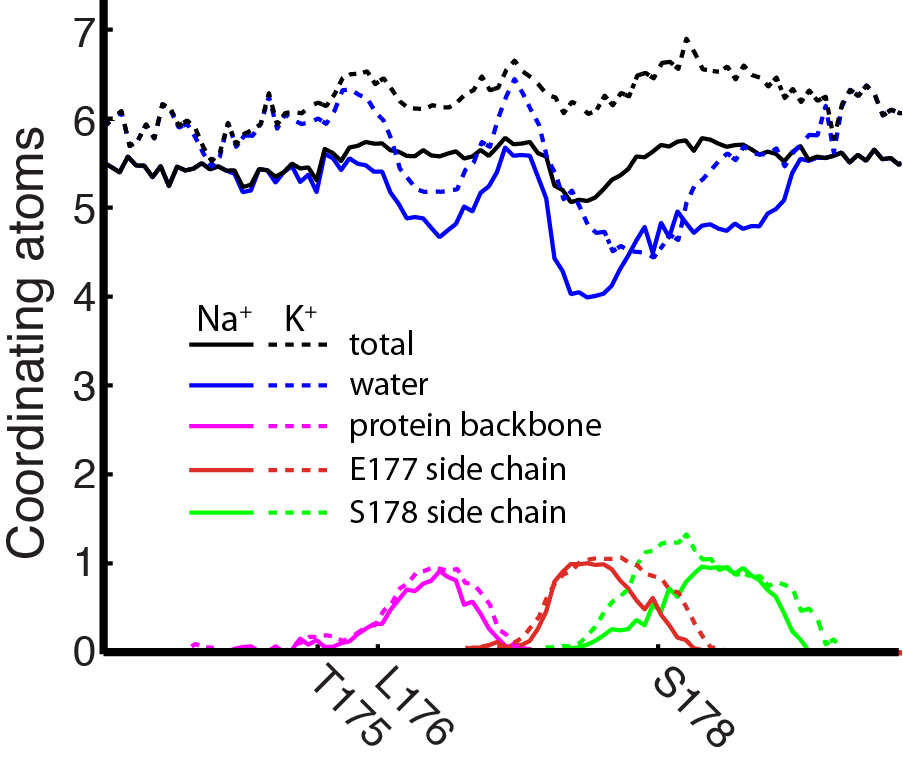

Supplement: Figure S2 — Coordination number of Na+ and K+ ions. Oxygen atoms were considered part of the coordination shell of the ion if closer than 2.8 Å from a Na+ ion or 3.2 Å from a K+ ion. All the umbrella-sampling trajectories from the simulations with two ions were used for this analysis, taking snapshots every 20 ps after the equilibration period. The black line shows the total number of coordinating oxygen atoms, which is the sum of the oxygen atoms coming from: water molecules (blue line), protein backbone (pink line), Glu177 side chain (red line), and Ser178 side chain (green line). The average positions along the filter axis of the carbonyl oxygen atoms of Thr175, Leu176, and Ser178 are shown along the x-axis. (TIF) [file pcbi.1002476.s002.tif]

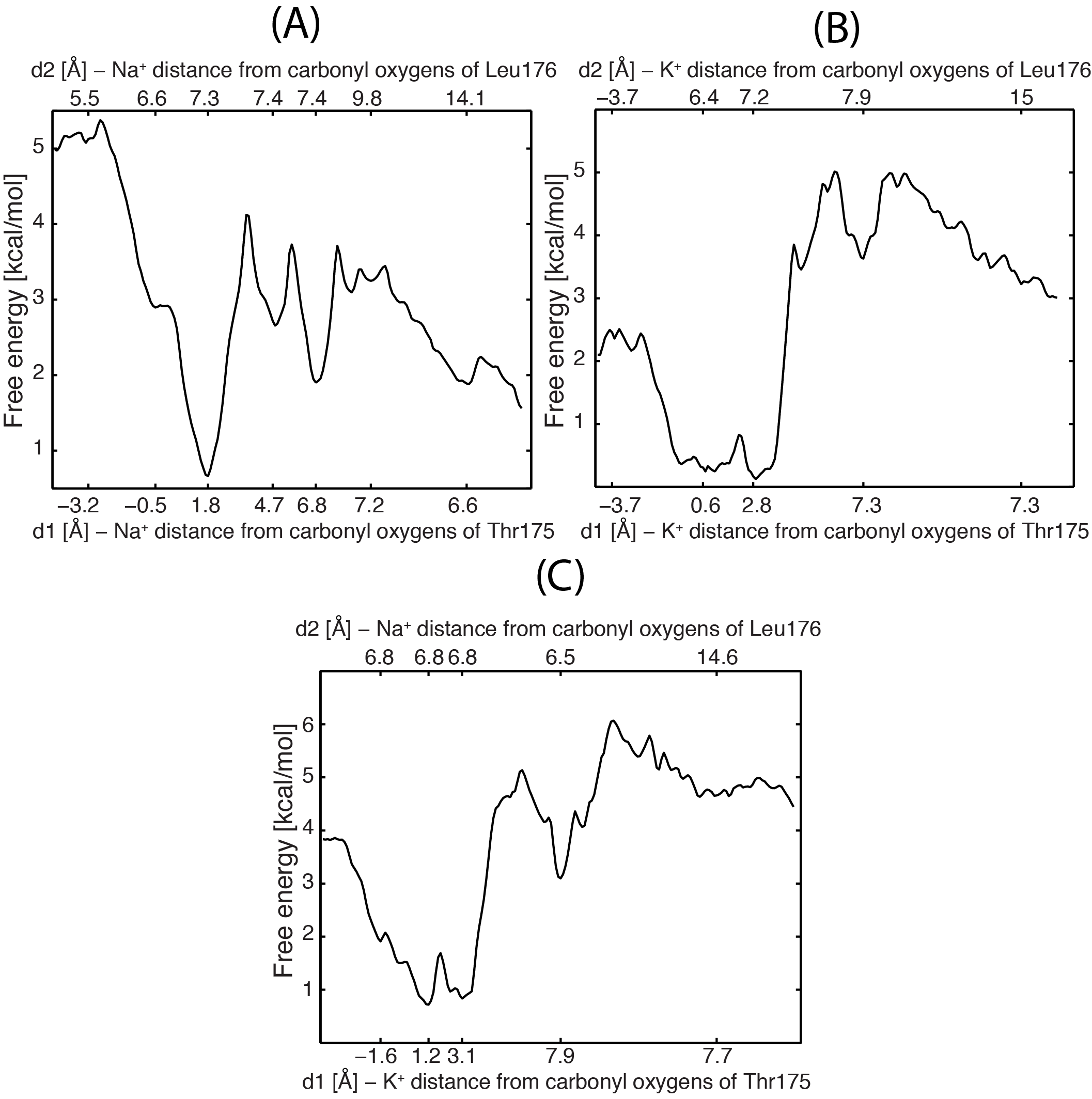

Supplement: Figure S3 — Free energy along the minimum energy path for Na+ ions (A), K+ ions (B), and K+/Na+ mixture with K+ in the innermost position (C). The minimum energy path was calculated between a local free-energy minimum with the bottom ion in the intracellular cavity and the top ion inside the selectivity filter, and a local free-energy minimum with the bottom ion inside the selectivity filter and the top ion in the extracellular solution. The path was discretized using 200 equally spaced points, and the minimum energy path was calculated using the string method. The values of the reaction coordinates, d1 and d2, along the minimum energy path are shown along the bottom and top x-axis for a subset of the local free-energy minima. d1/d2 are defined as the distance along the pore axis between the permeating ion in the inward/outward position and the centre of the carbonyl oxygen atoms of residues Thr175/Leu176. (TIF) [file pcbi.1002476.s003.tif]
